# Cytoplasmic PELP1 and ERRgamma Protect Human Mammary Epithelial Cells from Tam-Induced Cell Death

**DOI:** 10.1371/journal.pone.0121206

**Published:** 2015-03-19

**Authors:** Brian J. Girard, Tarah M. Regan Anderson, Siya Lem Welch, Julie Nicely, Victoria L. Seewaldt, Julie H. Ostrander

**Affiliations:** 1 Department of Medicine, Masonic Cancer Center, University of Minnesota, Minneapolis, Minnesota, 55455, United States of America; 2 Department of Medicine, Duke University Medical Center, Durham, North Carolina, 27710, United States of America; University of Tennessee Health Science Center, UNITED STATES

## Abstract

Tamoxifen (Tam) is the only FDA-approved chemoprevention agent for pre-menopausal women at high risk for developing breast cancer. While Tam reduces a woman's risk of developing estrogen receptor positive (ER+) breast cancer, the molecular mechanisms associated with risk reduction are poorly understood. Prior studies have shown that cytoplasmic proline, glutamic acid and leucine rich protein 1 (PELP1) promotes Tam resistance in breast cancer cell lines. Herein, we tested for PELP1 localization in breast epithelial cells from women at high risk for developing breast cancer and found that PELP1 was localized to the cytoplasm in 36% of samples. In vitro, immortalized HMECs expressing a nuclear localization signal (NLS) mutant of PELP1 (PELP1-cyto) were resistant to Tam-induced death. Furthermore, PELP1-cyto signaling through estrogen-related receptor gamma (ERRγ) promoted cell survival in the presence of Tam. Overexpression of ERRγ in immortalized HMECs protected cells from Tam-induced death, while knockdown of ERRγ sensitized PELP1-cyto expressing HMECs to Tam. Moreover, Tam-induced HMEC cell death was independent of apoptosis and involved accumulation of the autophagy marker LC3-II. Expression of PELP1-cyto and ERRγ reduced Tam-induced LC3-II accumulation, and knockdown of ERRγ increased LC3-II levels in response to Tam. Additionally, PELP1-cyto expression led to the upregulation of MMP-3 and MAOB, known PELP1 and ERRγ target genes, respectively. Our data indicate that cytoplasmic PELP1 induces signaling pathways that converge on ERRγ to promote cell survival in the presence of Tam. These data suggest that PELP1 localization and/or ERRγ activation could be developed as tissue biomarkers for Tam responsiveness.

## Introduction

Progress in breast cancer prevention is currently limited by our lack of biological markers to identify which women will respond to prevention therapies. Tamoxifen (Tam), a selective estrogen receptor modulator, is the most widely used treatment for estrogen receptor (ER)+ breast cancer. Tam treatment is approved for the prevention of breast cancer in pre-menopausal women, but it only reduces the risk of developing ER+ breast cancer by approximately 50% and does not prevent ER− breast cancer [1]. The increased risk of stroke, pulmonary emboli, cardiac events, endometrial cancer, and unwanted side effects (e.g., hot flashes, fatigue, depression, weight gain, and decreased libido) have decreased the acceptance of Tam among patients, particularly in the chemoprevention setting. Thus, there is a critical need to identify the women who are most likely to benefit from risk reducing strategies, and improve breast cancer prevention with novel prevention strategies. Inhibition of ER transcriptional activity is considered the predominate effect of Tam in invasive breast cancer; however, not all of Tam’s effects can be directly attributed to inhibition of ER. Tam is clinically effective in treatment of tumors that do not express ER [2]. Tam has a wide variety of ER-independent pharmacological activities including stimulation of transforming growth factor-beta, blockade of various chloride channels [3], inhibition of protein kinase C [4], and antagonism of calmodulin activity [5]. Additionally, Tam-binding sites independent of ER have been identified. Tam binds and regulates the G protein-coupled estrogen receptor (GPER) [6] and estrogen related receptors (ERRs) [7]. Furthermore, therapeutic concentrations of Tam are several orders of magnitude higher than the concentrations required to saturate ER [8]. On the basis of these observations, we hypothesized that ER-independent effects may play a role in Tam-induced cell death in normal or atypical breast tissue.

Members of the ERR subfamily of nuclear receptors (NRs) have been implicated in the ER-independent effects of Tam. ERR subfamily members include ERRα, ERRβ, and ERRγ. Although ERRs are considered orphan nuclear receptors with no known natural ligand, ERRβ and ERRγ have been shown to bind Tam [7,9,10]. ERRs are constitutively active transcription factors whose activity is predominately regulated through interactions with co-regulators. ERRs are primarily involved in the regulation of genes involved in cellular metabolism, energy homeostasis, and cancer [11]. While the role of ERRγ in breast cancer is relatively understudied, ERRγ expression has been associated with favorable breast cancer biomarkers, such as ER expression [12]. Conversely, ERRγ has been shown to promote Tam resistance in invasive ductal and lobular carcinoma cell culture models [13,14]. To date, a role for ERRγ in breast cancer initiation or response to Tam chemoprevention in mammary epithelial cell models has not been tested.

In addition to ERRγ, proline, glutamic acid and leucine-rich protein-1 (PELP1), a nuclear receptor co-activator protein, has been shown to promote Tam resistance in invasive breast cancer cell line models. Most co-activators function in the nucleus to enhance the transcriptional activation function of nuclear receptors (NRs), but PELP1 has been shown to regulate genomic and extra-nuclear (cytoplasmic) actions [15,16]. *In vivo*, PELP1 subcellular localization is primarily nuclear in normal breast tissue, but it is localized to the cytoplasm in about 40% of invasive breast tumors [17]. Targeting PELP1 to the cytoplasm by mutation of the nuclear localization signal (PELP-cyto) leads to activation of non-genomic signaling and Tam resistance in breast cancer cell line models [17]. Interestingly, in a mammary-specific transgenic mouse model, expression of PELP1-cyto induced mammary gland hyperplasia that was unaffected by Tam treatment [18]. Although PELP1 is localized to the cytoplasm in about 40% of invasive breast cancers, PELP1 localization in pre-malignant or high-risk tissues is not known. We therefore hypothesized that cytoplasmic PELP1 drives breast cancer initiation and epithelial cell survival though an ER-independent mechanism involving ERRγ. Upregulation of this pathway may explain resistance to Tam chemoprevention. Herein, we investigated the role of PELP1 and ERRγ in promoting cell survival in response to Tam in ER− models of early mammary carcinogenesis.

## Materials and Methods

### Subjects and Informed Consent

The study was approved by the Human Subjects Committee and Institutional Review Board at Duke University Medical Center in accordance with assurances filed with and approved by the Department of Health and Human Services. Subjects were recruited on entry to the Duke University High-Risk Clinic and were required to have one of the following: (a) a 5-year Gail model risk score ≥1.7%, (b) a prior biopsy exhibiting atypia, LCIS, or DCIS, or (c) known or suspected BRCA1/2 mutation. Women in the Duke High-Risk Clinic were initially approached by Dr. V.L. Seewaldt or her physician assistant for consent to random periaeriolar fine needle aspiration (RPFNA). A study nurse or coordinator then obtained written consent by participants signing an IRB-approved consent form. The Duke High-Risk Research Cohort was established in 2003. Currently there are 1027 women participating in the Duke High-Risk Research Cohort (defined as all women participating in research studies). The protocol and consent has undergone yearly review and all investigators have completed full Duke human subjects, ethics, and compliance training.

### PELP1 localization in RPFNA samples

RPFNA samples were prepared for immunofluorescence at the Duke University Medical Center Cytology Laboratory by ThinPrep. Immunofluorescence staining on all 11 samples was performed together. Pan-cytokeratin staining was used to identify epithelial cells and total cell region. DAPI staining was used to define the nuclear region. PELP1 subcellular localization was quantified by using ImageJ software. Briefly, PELP1 fluorescence intensity of the cytoplasmic compartment was divided by the total PELP1 fluorescence intensity of each individual epithelial cell. Epithelial cells that contained at least 50% of total PELP1 in the cytoplasm were considered positive for cytoplasmic PELP1. Three independent cell clusters from each sample were analyzed for PELP1 localization on a cell-by-cell basis. PELP1 was considered to be predominately cytoplasmic if ≥50% of total PELP1 expression was observed in at least 20% of analyzed cells.

### Cell culture and reagents

MCF-10A and MCF-7 cells were purchased from American Type Culture Collection. HMEC-hTERT mammary epithelial cells were obtained from Lonza as primary cells and immortalized as previously described [19]. 240Lp16sMY mammary epithelial cells were a generous gift from Martha Stampher (Lawrence Berkeley National Laboratory) and have been described previously [20]. MCF-7 cells were cultured in minimal essential medium α (Invitrogen) supplemented with 5% fetal bovine serum, 10 mmol/L HEPES (Invitrogen), 1× nonessential amino acids (Invitrogen), 1× sodium pyruvate (Invitrogen), 1 μg/mL insulin (Invitrogen), 1 μg/mL hydrocortisone (Sigma Aldrich), and 10 ng/mL epidermal growth factor (EGF; Sigma Aldrich). HMEC-hTERT cells were cultured in HuMEC Ready Media (1X) (Life Technologies) supplemented with epidermal growth factor, hydrocortisone, isoproterenol, transferrin, insulin, and 25 mg of bovine pituitary extract. MCF-10A [21] and 240Lp16sMY [22] cells were cultured as described previously.

### Generation of PELP1 and ERRγ Cell Lines

Full length PELP1 cDNA was generated by RT-PCR from RNA isolated from MCF-7 cells. The primers used to amplify PELP1 were 1) 5’-GAAGATGGCGGCAGCCGTTCT-3’ and 2) 5’-GTGGGGTGCAGAAGATGGCT-3’. The amplified product was gel purified and ligated into pCR-2.1. pCR-2.1-PELP1 cDNA was sequenced and compared to NCBI Reference Sequence NM_014389.2. Any base-pair changes were converted to match the reference sequence by site-directed mutagenesis. PELP1 encoding a mutation in the nuclear localization signal (KKLK → EELE), termed PELP1-cyto was made by site-directed mutagenesis of PELP1 wild-type (wt), as previously described [17]. PELP1-wt and PELP1-cyto were digested out of pCR2.1 and subcloned in to pLXSN for retrovirus production. HMEC-hTERT and 240Lp16sMY cells were transduced with retrovirus encoding pLXSN vector control, PELP1-wt, or PELP1-cyto. Cells were selected with 500 μg/mL of G418, and single-cell cloning was used on selected cells to generate clonal cell lines overexpressing the respective PELP1 cDNA. PELP1-wt and PELP1-cyto expression was confirmed by Western blot and immunofluorescence for PELP1.

We obtained 6 ERRγ shRNA target constructs (pLKO.1) from The RNAi Consortium shRNA Library. Lentivirus for all 6 shRNA was generated and knockdown tested in multiple cell lines after lentiviral infection, and stable pooled populations were selected with 0.5 ug/mL puromycin. ERRγ mRNA transcript levels were tested by qRT-PCR. The target shRNA sequence that achieved the best knockdown was 5’-CCTGTCAGGAAACTGTATGAT-3’. We used this shRNA for all subsequent knockdown experiments. To achieve knockdown in HMEC-hTERT cell lines, we subcloned the shRNA into pLKO.1-Hygro by annealing 50 basepair oligos with suitable overhangs for inserting into this vector. Lentivirus was then produced, and cells were infected with the ERRγ targeting vector or shRNA control. Stable pooled populations were selected for with 50 μg/mL hygromycin. To achieve overexpression of ERRγ in our cell lines, we purchased the lentiviral overexpression vector, pEZ-Lv152, containing a complete cDNA for ERRγ with control (GeneCopoeia, EX-E3315-Lv152). We validated the constructs by transfection into cells and measuring ERRγ mRNA transcript levels by qRT-PCR. Once validated, we generated lentivirus to make stable pools overexpressing ERRγ, which were selected with 50 μg/mL of hygromycin and maintained in 20 μg/mL hygromycin.

### Western blotting and antibodies

Whole-cell lysates were collected using RIPA buffer [10 nm sodium phosphate (pH 7.0), 150 mm NaCl, 2 mm NaCl, 2 mm EDTA, 1% (wt/vol) Nonidet P-40 (NP-40), 0.1% (wt/vol) SDS, 1% sodium deoxycholate, 0.1% (vol/vol) β-mercaptoethanol, supplemented with 20 μg/mL aprotinin, 0.1 m sodium fluoride, 1 mm sodium vanadate, 1 mm phenylmethylsulfonyl fluoride] and scraping on ice. Nuclear and cytoplasmic fractions were collected using the NE-PER Nuclear Protein Extraction Kit (Thermo Scientific). Lysates were centrifuged at 14,000 rpm at 4°C, and supernatant was collected. A BCA Assay (Thermo Scientific) was performed for protein concentration quantification, and lysates were resolved on an SDS-PAGE gel. Proteins were transferred onto a polyvinylidene difluoride membrane and blocked in 5% BSA in TBST for 1 hour. Membranes were subsequently incubated in primary antibody (1–2 hours at room temperature or overnight at 4°C), washed, and then incubated with an HRP-conjugated secondary antibody for 1 hour at room temperature. Antibody-protein interactions were visualized using SuperSignal West Pico Chemiluminescent Substrate (Thermo Scientific) or Immobilon Western Chemiluminscent HRP Substrate (Millipore) according to the manufacturer's protocol. The following antibodies were used: PELP1 (A300–180A, Bethyl Laboratories, Inc.), MAP1LC3A/B (AHP2167, AbD Serotec), Actin-HRP, HDAC2, and p65 (sc-1616, sc-7899, sc-8008, Santa Cruz Biotechnology, Inc. respectively).

### Flow Cytometry

For cell cycle analysis, cells were plated at a density of 100,000 cells per well into 6-well plates. Twenty four or 48 hours after treatment with Tam or nocodozole (positive control), cells were harvested in StemPro Accutase Cell Dissociation Reagent (Life Technologies). Cells were rinsed with cold PBS and resuspended in 500 μL of staining solution as previously described[23]. Cells were incubated for 15 minutes and filtered through FACS tubes with a cell-strainer cap. Samples were then analyzed on a BD LSR II flow cytometer to measure propidium iodide incorporation. Data was analyzed in FlowJo and cell cycle analysis was carried out manually and using the Watson (Pragmatic) algorithm.

For Annexin V/7-AAD staining, cells were plated at a density of 100,000 cells per well into 6-well plates. Cells were treated the next day with Tam. Twenty four hours after treatment, cells were harvested using StemPro Accutase Cell Dissociation Reagent to preserve membrane integrity. Cells were then processed using PE Annexin V Apoptosis Detection Kit I (BD Pharmingen) and subsequently analyzed in a FACSCalibur flow cytometer to measure Annexin V and 7-AAD staining. Data was analyzed using FlowJo software.

### MTT Assay

Cells were plated at 5,000 cells per well into 1 mL of growth media in 24-well dishes. A treatment day reading was taken to ensure even cell plating density. Cells were treated with Tam or Tam plus inhibitors in HuMEC medium one day after plating. Cell viability was measured 3 days after treatment. Viability was assayed by the uptake of MTT as described previously [24].

### RNA isolation, cDNA synthesis, and Q-RT-PCR

RNA extraction was carried out using TriReagent (Life Technologies) according to the manufacturer’s protocol. RNA concentration and purity were determined by using a Biotek Synergy 2 microplate reader. The 260/280 ratio of all samples used was ≥ 1.7. cDNA synthesis was carried out using 1 μg of RNA and qScript cDNA SuperMix (Quanta BioSciences) according to the manufacturer’s protocol. Real-time quantitative PCR analysis was performed on a Light Cycler (Roche Diagnostics) according to the manufacturer's instructions. LightCycler 480 SYBR Green 1 Master (Roche) was used to detect double stranded DNA. Cumulative fluorescence was measured at the end of the extension phase of each cycle, and cycle numbers obtained at the log-linear phase of the reaction were plotted against a standard curve prepared with serially diluted samples for relative quantification of mRNA abundance. Results were normalized to either β-actin, GAPDH, or TBP-2, which served as housekeeping genes.

## Results

### Cytoplasmic PELP1 localization in RPFNA samples from high-risk women

To determine whether PELP1 localization is altered during early mammary carcinogenesis, we examined PELP1 localization in 11 randomly selected RPFNA samples from asymptomatic women at high risk for developing breast cancer. Needle aspirates were fixed as previously described [25] and prepared for immunofluorescence (IF) by ThinPrep. All samples were incubated with primary antibodies to PELP1 and pan-cytokeratin, followed by incubation with secondary antibodies and DAPI counterstaining. Epithelial clusters were imaged, and PELP1 localization was quantitated using ImageJ software (see Materials and Methods). We found that 4/11 (36%) RPFNA samples were positive for cytoplasmic PELP1 staining. Cytoplasmic PELP1 expression ranged from 5% to 70% of all epithelial cells (Table 1). Only samples that had at least 20% of the total population expressing cytoplasmic PELP1 were considered positive. These results indicate that PELP1 localization is altered in mammary epithelial cells from high-risk women and altered PELP1 localization may be an early event in breast cancer initiation.

**Table 1:** PELP1 localization in RPFNA samples.

Sample #	% of cells with cytoplasmic PELP1
**1**	**58.1**
2	0.0
3	3.2
4	0.0
**5**	**25.0**
6	1.4
7	7.7
8	2.9
**9**	**37.5**
**10**	**27.5**
11	1.7

### Immortalized HMECs are sensitive to Tam

**Fig 1:**
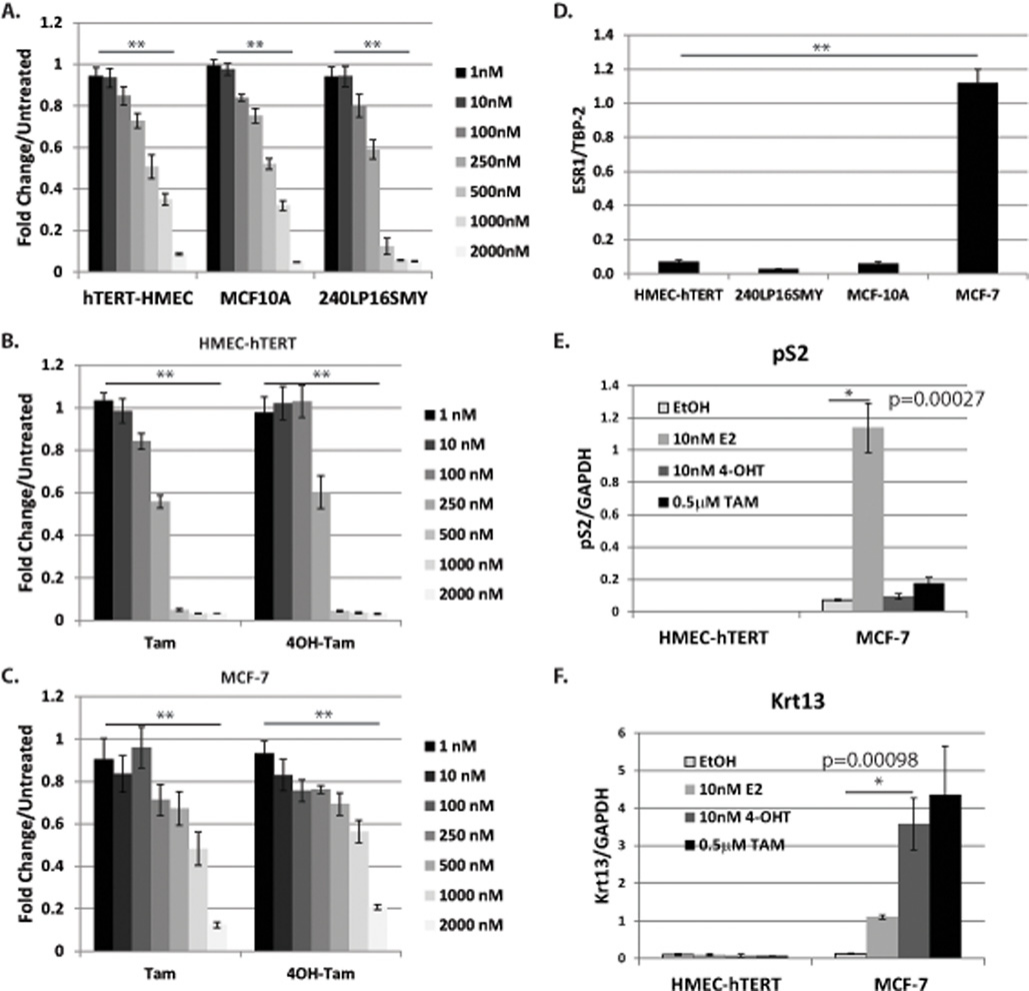
Human mammary epithelial cell lines are sensitive to Tam independent of ER expression. **A**, MTT assay of mammary epithelial cell lines treated with increasing concentrations of Tam for 3 days. MTT assay of HMEC-hTERT (**B**) and MCF-7 (**C**) cell lines treated with increasing concentrations of either Tam or 4-OH-Tam for 3 days. **D**, qRT-PCR of ESR1 and TATA-binding protein 2 (TBP2) (internal control) mRNA levels in three mammary epithelial cell lines and MCF-7 cells. HMEC-hTERT and MCF-7 cells were treated with ethanol (control), 10 nM E2, 10 nM 4-OH-Tam, or 0.5 μM Tam for 24 hours. ER target genes pS2 (**E**) and Krt13 (**F**) were measured by qRT-PCR and averaged over values for glyceraldehyde-3-phosphate dehydrogenase (*GAPDH*) (internal control). A student’s T-Test was performed to determine statistical significance for qRT-PCR data. P-values are given for the indicated comparisons. A-D) Oneway ANOVA was performed to determine statistical significance of presented data. ** indicates p < 0.001.

We and others have shown that ER-low or ER-negative cell lines are responsive to Tam at physiological concentrations [26–32]. Prior to testing the effect of PELP1-cyto expression on Tam responsiveness, we examined Tam sensitivity among a panel of HMECs: hTERT immortalized HMECs (HMEC-hTERT), immortalized 240Lp16sMY cells (p16 knockdown, c-myc overexpression), and spontaneously immortalized MCF-10A cells. All cell lines were plated in growth medium and treated the following day with human mammary epithelial cell growth medium (HuMEC, Life Technologies) containing Tam. MTT assays were performed three days following Tam treatment. We found that all cell lines tested were sensitive to Tam starting at 100 nM with increasing cell death from 250 nM to 2 μM (Fig. 1A). Of note, we did observe some experimental variability in the Tam dose needed to achieve 50% cell death; ranging from 250 nM to 750 nM. Therefore, all subsequent MTT experiments tested at least two concentration of Tam.

To determine whether the effect of Tam in HMECs was independent of ER action, we compared the effects of Tam and 4-hydroxytamoxifen (4-OHT) in HMEC-hTERT (ER−) and MCF-7 (ER+) cells. Tam is taken orally as tamoxifen citrate and then metabolized *in vivo* to 4-OHT, which binds with higher specificity to ER [33]. Tam and 4-OHT treatment both resulted in significant cell death at a concentration of 0.5 μM in HMEC-hTERT cells (Fig. 1B). Interestingly, while MCF-7 cells were sensitive to Tam at the same concentrations, the effect in HMECs was more robust (Fig. 1B and 1C). This is likely because of ER-dependent growth arrest in MCF-7 cells [34] versus cytotoxic cell killing of HMECs. We also examined ESR1 mRNA expression in our HMEC lines and MCF-7 cells. As expected, very little mRNA for ESR1 was found in HMEC-hTERT, 240Lp16sMY, or MCF-10A cells, while MCF-7 cells expressed appreciable levels of ESR1 (Fig. 1D). To determine if ER-dependent gene expression was modulated in HMEC-hTERT cells, HMEC-hTERT and MCF-7 cells were treated with 17β-estradiol (E2), 4-OHT, and Tam for six hours, and cDNA was prepared from isolated RNA. qRT-PCR was then performed to examine levels of pS2 and Krt13. pS2 is a known E2-induced, ER-response gene, and Krt13 is an ER-dependent, 4-OHT-induced gene [35]. As expected, pS2 and Krt13 were induced by E2 and 4-OHT, respectively, in MCF-7 cells, but no regulation of these genes was observed in HMEC-hTERT cells (Fig. 1E and 1F). Together, these data suggest that Tam-induced cell death of immortalized HMECs is independent of ER expression.

### Expression of PELP1-cyto inhibits Tam-induced cell death

**Fig 2:**
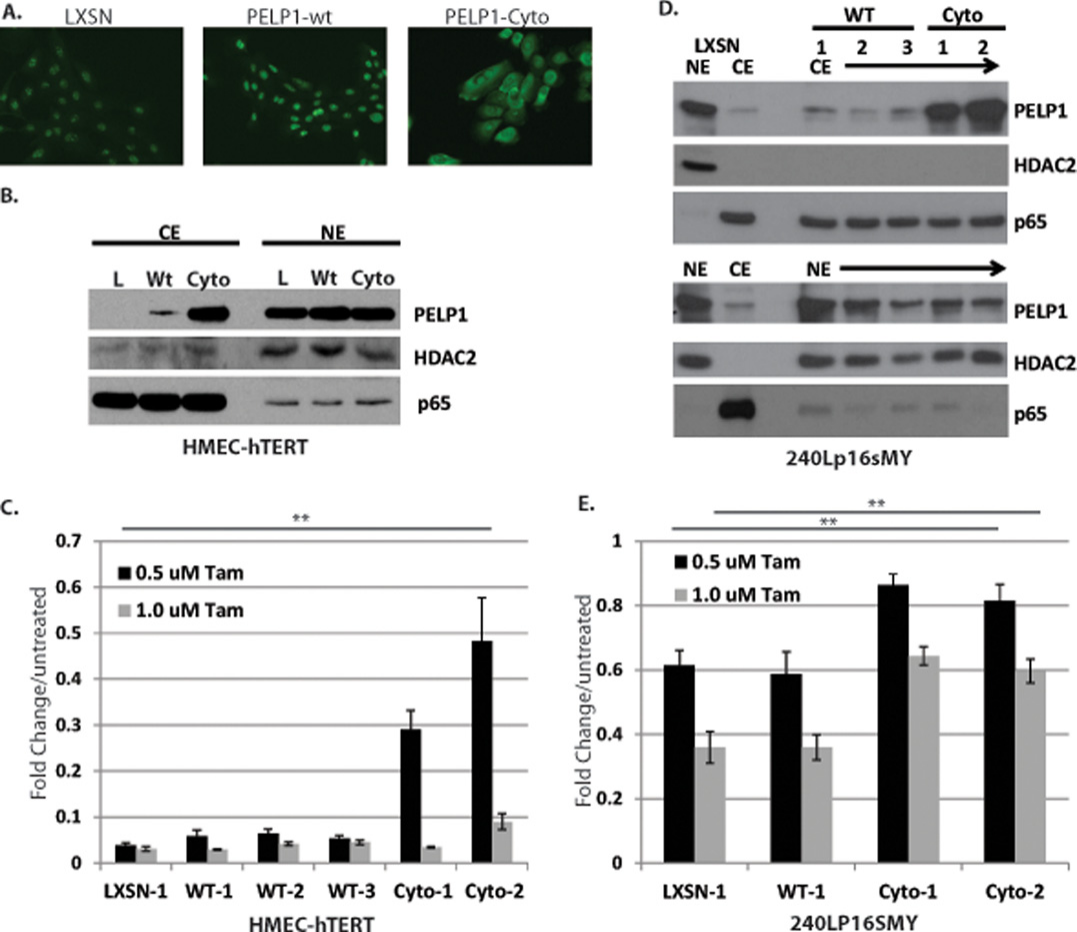
Cytoplasmic PELP1 protects HMECs from Tam-induced cell death, independent of Akt and Erk1/2. **A**, Immunofluorescence of HMEC-hTERT cells stably expressing vector control (pLXSN), PELP1-wild type (wt), or PELP1-cyto. HMEC-hTERT (**B**) and 240Lp16sMY (**D**) cell lines stably expressing vector control (pLXSN), PELP1-wild-type (wt), or PELP1-cyto were examined by Western blotting of nuclear (NE) and cytoplasmic (CE) fractions with antibodies against PELP1, HDAC2, and p65., MTT assay of HMEC-hTERT (**C**) and 240Lp16sMY (**E**) cell lines expressing pLXSN, PELP1-wt, or PELP1-cyto were treated with 0.5 or 1.0 μM Tam for 3 days. One-way ANOVA was performed to test for statistical differences between cell lines treated with 0.5 μM Tam (HMEC-hTERT) and 0.5 μM and 1.0 μM Tam (240Lp16sMY). ** indicates p < 0.0001.

To determine whether PELP1 localization has an effect on Tam-induced cell death in immortalized HMECs, we established stable cell lines that express vector control (pLXSN), PELP1-wild-type (wt), or PELP1-cyto (NLS mutant). Cells were selected for stable integration of PELP1 with G418. Clonal cell populations were screened for PELP1 localization by immunofluorescence (Fig. 2A) and Western blotting of cytoplasmic and nuclear fractions (Fig. 2B and 2D). Clonal cell lines expressing PELP1-cyto showed increased PELP1 in the cytoplasm compared to PELP1-wt and vector control cell lines. Western blotting for p65 and HDAC2 was performed as controls for cytoplasmic and nuclear fractionation, respectively (Fig. 2B and 2D). Vector control, PELP1-wt, and PELP1-cyto cells were tested for response to Tam by MTT assay (as described in Materials and Methods). HMEC-hTERT and 240Lp16sMY clonal cell lines expressing PELP1-cyto, but not PELP1-wt or vector control, were more resistant to Tam-induced cell death (Fig. 2C and 2E).

**Fig 3:**
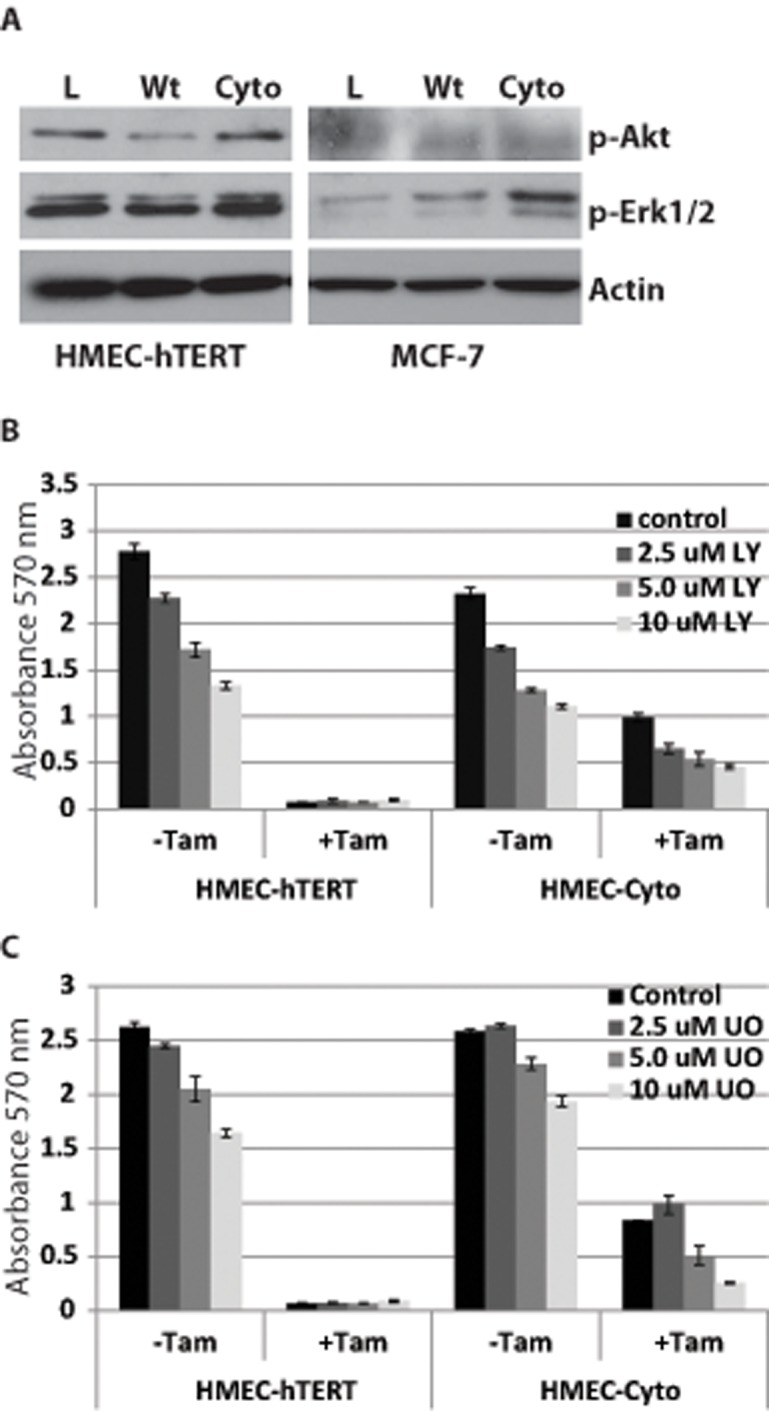
PELP1-cyto does not activate Akt and Erk1/2 to promote cell survival of HMECs in the presence of Tam. **A**, Western blotting for phospho-Akt and phospho-Erk1/2 in HMEC-hTERT and MCF-7 cells. Actin served as a loading control. **B** and **C**, MTT assay of HMECs expressing pLXSN or PELP1-cyto were treated with increasing concentrations of the Akt inhibitor LY294002 (LY) (**B**) or the MAPK inhibitor UO126 (UO)(**C**). The vehicle for LY and UO was DMSO.

Prior studies in ER+ breast cancer cell line models have shown that PELP1-cyto promotes activation of ERK1/2 and Akt signaling through EGFR to promote resistance to Tam [17,18]. To determine if similar signaling pathways contributed to PELP1-cyto-induced cell survival in response to Tam in HMEC-hTERT cells we examined PELP1-cyto-induced effects on phosphorylation of Akt Ser473 and Erk1/2 Thr202/Tyr204 by Western blotting of whole cell extracts. Interestingly, we did not observe robust PELP1-cyto-, or PELP1-wt-, induced phosphorylation of Akt or Erk1/2 in HMEC-hTERT cells. We did observe increased phosphorylation of Erk1/2 in MCF-7 cells expressing PELP1-wt and PELP1-cyto compared to MCF-7 LXSN control cells. Phosphorylation of Akt was below our detection limits in MCF-7 cells (Fig. 3A). To determine whether inhibition of these pathways sensitized PELP1-cyto cells to Tam-induced death, HMECs were treated with increasing concentrations of the PI3K inhibitor LY294002 (LY) or the MEK1 inhibitor UO126 (UO) in the presence or absence of 0.5 μM Tam (Fig. 3B and 3C). Both LY and UO had a modest effect on the growth of HMEC-hTERT control and PELP1-cyto HMECs in the absence of Tam. Neither drug, however, sensitized PELP1-cyto expressing HMECs to Tam-induced cell death when compared to vehicle (DMSO) treated cells. These data suggest that the mechanism of PELP1-cyto-induced cell survival in response to Tam is largely independent of Akt and Erk1/2 signaling in HMECs.

### ERRγ expression modulates response to Tam in HMECs

**Fig 4:**
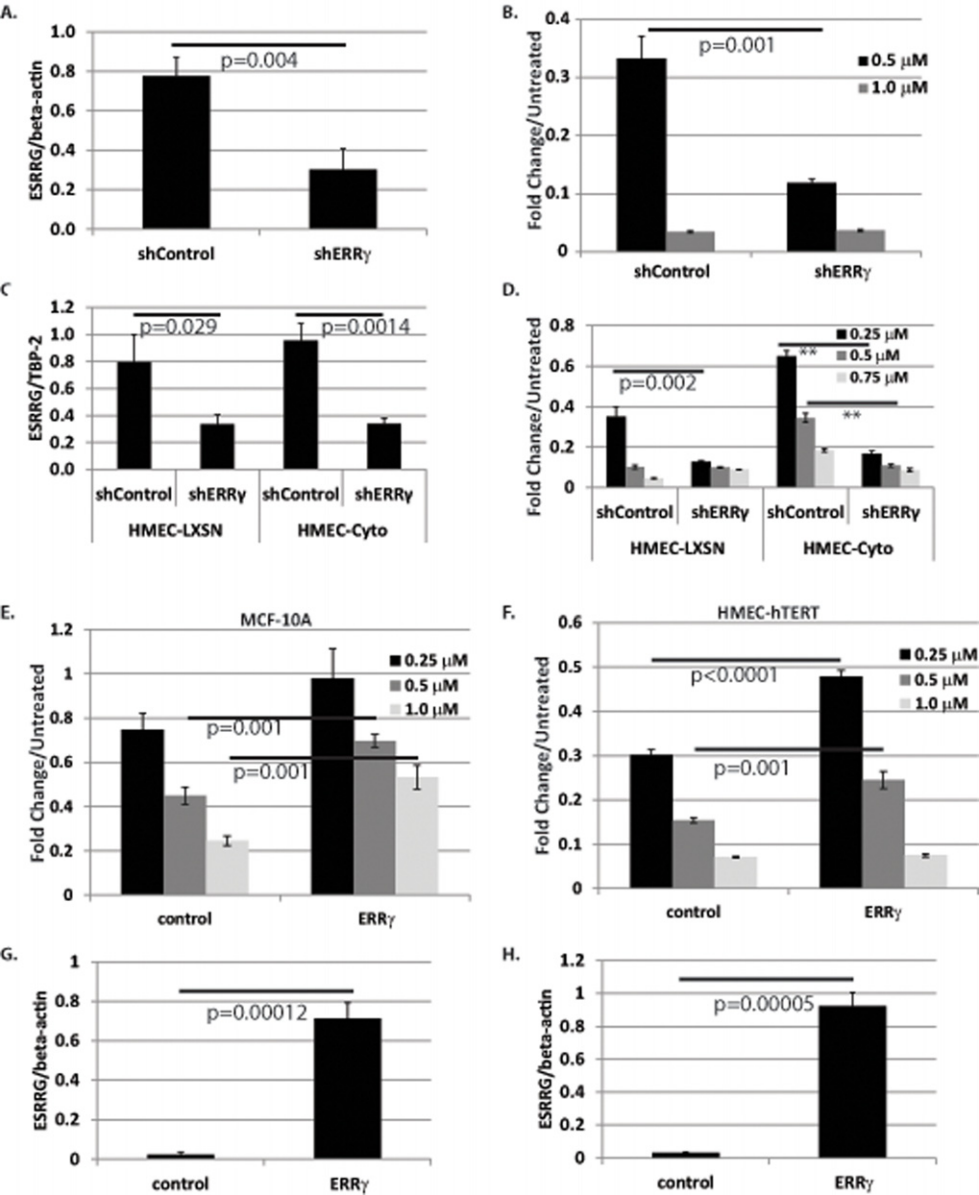
Knockdown and overexpression of ERRγ alters response to Tam in HMECs. **A**, qRT-PCR for ERRγ mRNA to verify knockdown in shControl and shERRγ stable cell lines. **B**, MTT assay of MCF-10A cell lines expressing either shRNA control (shControl) or shRNA against ERRγ (shERRγ). Cells were treated with 0.5 or 1.0 μM Tam for 3 days. **C**, qRT-PCR for ERRγ mRNA to verify knockdown in HMEC-LXSN and HMEC-Cyto cell lines stably expressing either shControl or shERRγ. **D**, MTT assay of HMEC-LXSN and HMEC-Cyto expressing either shControl or shERRγ. Cells were treated with increasing concentrations of Tam for 3 days. **E**, MTT assay of MCF-10A cell lines expressing vector control or ERRγ. Cells were treated with increasing concentrations of Tam. **F**, MTT assay of HMEC-hTERT cells stably expressing vector control or ERRγ. Cells were treated with increasing concentrations of Tam for 3 days. ERRγ overexpression in MCF-10A (**G**) and HMEC-hTERT (**H**) cell lines was verified by qRT-PCR of ERRγ. One-way ANOVA was performed to test for statistical differences between cell lines treated with Tam in MTT assays. Student’s T-test was performed to determine statistical significance for qRT-PCR experiments. P-values are shown for the indicated comparisons. ** indicates p<0.0001.

Tam modulates intracellular signaling in cell line models through a variety of pathways (reviewed in [36]). Tam binds ERRγ [37], and ERRγ promotes Tam resistance in ER+ breast cancer cell line models [13,14,38]. To determine whether ERRγ expression affects response to Tam, we first knocked down ERRγ in MCF10A cells. MCF10A cells were infected with lentivirus encoding control shRNA (pLKO.1 shGFP) and ERRγ shRNA. Cells were selected for stable incorporation of the shRNA with puromycin. Pooled cells were tested for ERRγ expression by qRT-PCR to confirm ERRγ knockdown (Fig. 4A). MCF10A cells positive for ERRγ knockdown were more sensitive to 0.5 μM Tam compared to shRNA control cells (Fig. 4B, p = 0.001). Next, we knocked down ERRγ in vector control and PELP1-cyto HMEC-hTERT cells. After lentiviral transduction with virus encoding control shRNA or ERRγ shRNA, infected cells were selected for stable incorporation of the shRNA with hygromycin. Pooled populations were tested for ERRγ knockdown (Fig. 4C) and response to Tam by MTT. Similar to what we observed in MCF-10A cells, control HMEC-hTERTs expressing ERRγ shRNA were sensitized to Tam at 0.25 μM Tam (Fig. 4B, p = 0.002). Additionally, resistance to Tam treatment was lost in HMEC-hTERT-Cyto cells expressing ERRγ shRNA at 0.25 and 0.5 μM Tam (Fig. 4D, p<0.0001).

To complement our ERRγ knockdown results, ERRγ was overexpressed in HMEC-hTERT and MCF-10A cells. Parental HMEC-hTERT and MCF10A cells were infected with lentivirus encoding vector control or ERRγ. Cells were selected for stable integration of ERRγ with hygromycin. As expected, ERRγ overexpression protected both HMEC-hTERT and MCF-10A cells from Tam-induced cell death compared to vector control expressing cells, particularly at 0.5 and 1.0 μM Tam (Fig. 4E, p = 0.001) in MCF-10A cells, and 0.25 and 0.5 μM Tam in HMEC-hTERT cells (Fig. 4F, p<0.001 and p = 0.001). Overexpression of ERRγ mRNA in pooled populations was confirmed by qRT-PCR (Fig. 4G and 4H). Of note, we relied on mRNA expression of ERRγ to confirm knockdown and overexpression because all ERRγ antibodies that we tested did not specifically recognize ERRγ (an HA-tagged ERRγ construct was utilized to test all antibodies and then confirm ERRγ expression with an HA antibody). These data indicate that cytoplasmic PELP1-mediated resistance to Tam-induced cell death is dependent on ERRγ expression.

### PELP1-cyto signaling and ERRγ regulated gene expression

**Fig 5:**
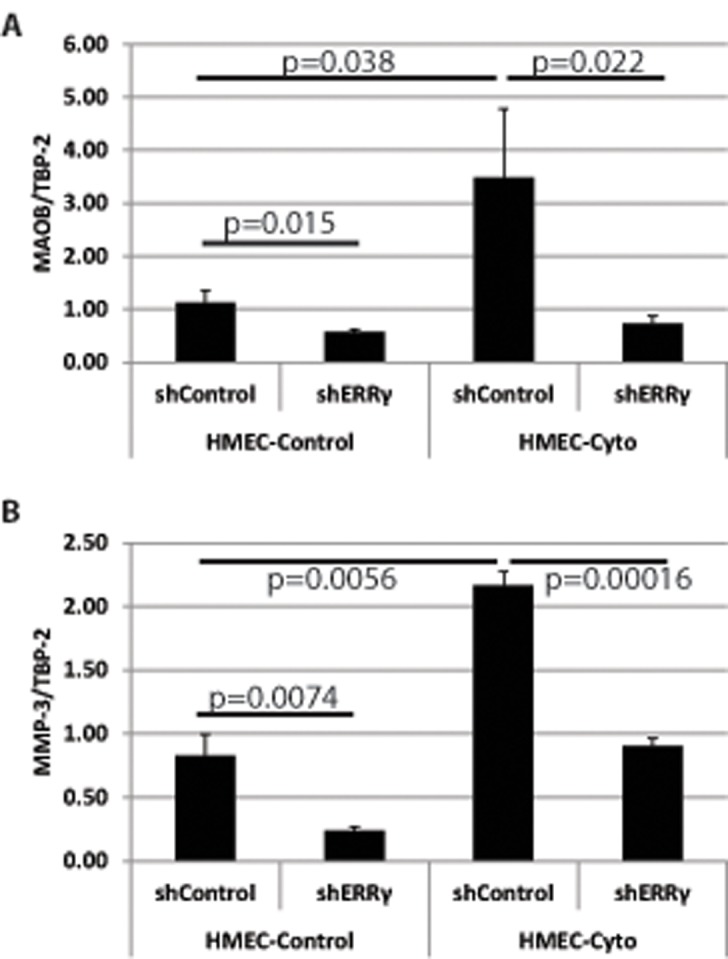
Knockdown of ERRγ regulates PELP1-cyto genes. MAOB (**A**) and MMP-3(**B**) were measured by qRT-PCR in HMECs expressing pLXSN or PELP1-cyto and shControl or shERRγ. A student’s T-Test was performed to determine statistical significance for qRT-PCR experiments; p-values are shown for the indicated comparisons.

To determine whether PELP1-cyto signaling modulates ERRγ-dependent gene expression, we examined expression of MAOB and MMP-3, genes previously identified to be regulated by ERRγ or PELP1, respectively. RNA was isolated from HMEC-hTERTs (control and PELP1-cyto) expressing either control shRNA or ERRγ shRNA. We found that MAOB, a classical ERRγ target gene [39], was upregulated in PELP1-cyto expressing HMECs. Furthermore, shRNA knockdown of ERRγ inhibited PELP1-cyto-induced upregulation of MAOB (Fig. 5A). We found that MMP-3 expression, which is regulated by PELP1 in MDA-231 breast cancer cells [40], was upregulated in PELP1-cyto HMECs. Similar to MAOB, knockdown of ERRγ inhibited PELP1-cyto-induced MMP-3 expression (Fig. 5B). These data suggest that PELP1-cyto mediated signaling events modulate ERRγ transcriptional activity.

### PELP1-cyto and ERRγ protect HMECs from Tam-induced autophagy-associated cell death

**Fig 6:**
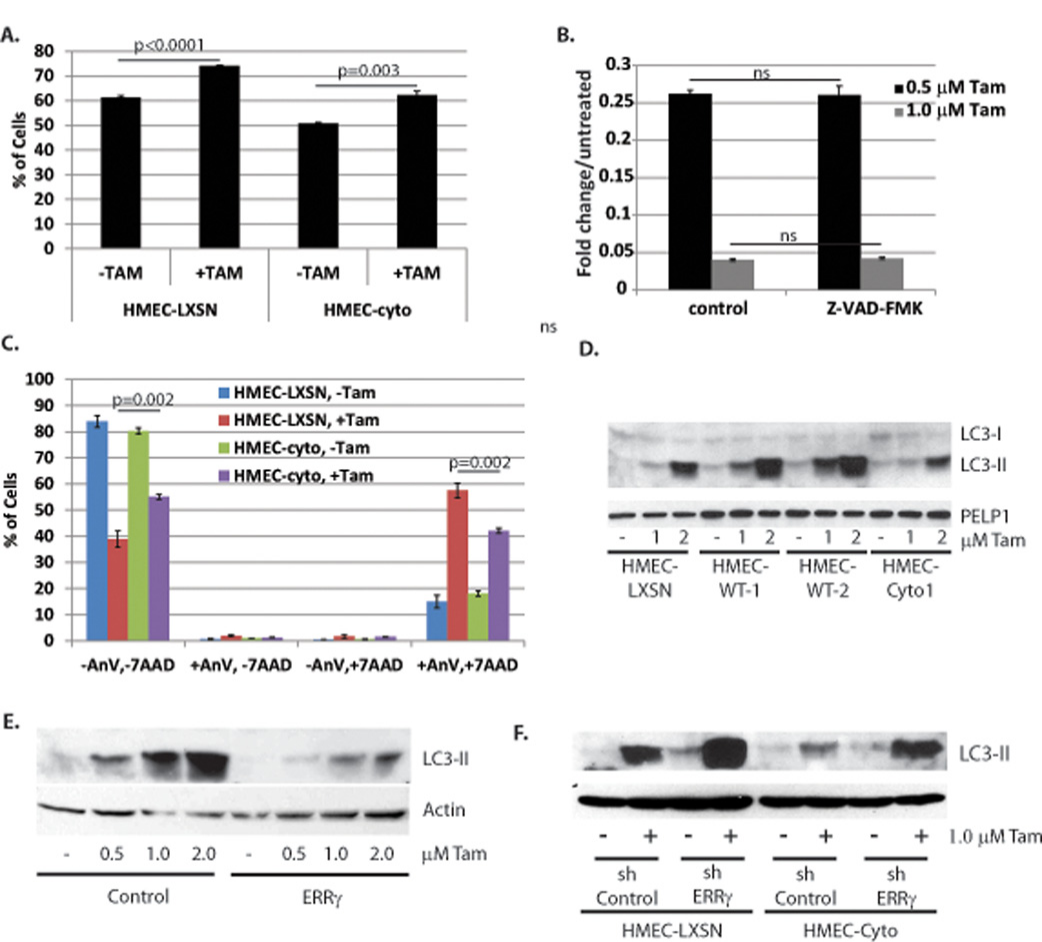
Cytoplasmic PELP1 and ERRγ protect cells from Tam-induced autophagy. **A**, Cells in G1/G0 from cell cycle analysis of propidium iodide stained HMEC-LXSN and HMEC-Cyto cells treated with vehicle control or 1.0 μM Tam for 24 hours. A student’s T-Test was performed to determine statistical significance for changes in G1/G0 cell populations in response to Tam. P-values are given for the indicated comparisons. **B**, MTT assay of HMEC-hTERT cells treated with 0.5 or 1.0 μM Tam and ethanol control or 25 μM Z-VAD-FMK. A student’s T-Test was performed to determine statistical significance between control and Z-VAD-FMK treated cells in the presence of Tam. “ns” indicates no statistically significant differences were observed. **C**, Annexin V/7-AAD staining of HMEC-LXSN and HMEC-Cyto cells treated with 1.0 μM Tam for 24 hours. Student’s T-test was performed to determine statistical significance between Tam treated HMEC-LXSN and HMEC-cyto cells; p-values are shown for the indicated comparisons. **D**, Western blotting for LC3-I and LC3-II from HMEC-LXSN, HMEC-WT-1/WT-2, and HMEC-cyto lysates treated with ethanol control, 1 μM Tam, or 2 μM Tam for 48 hours. **E**, Western blot for LC3-II of whole cell lysates from HMECs expressing control or ERRγ overexpression vector, treated with control or 0.5, 1, or 2 μM Tam for 24 hours. **F**, Western blot for LC3 from whole cell lysates of HMEC-LXSN and HMEC-cyto cells expressing either shControl or shERRγ, treated with ethanol control or 1 μM Tam for 24 hours.

Our data indicate that PELP1-cyto and ERRγ are involved in an ER-independent molecular mechanism of Tam resistance. Tam has been shown to induce cell cycle arrest, apoptosis, and autophagy. To understand the mechanism of Tam-induced death in our *in vitro* HMEC models, we examined the biological effects of Tam on HMEC-control and HMEC-cyto cells. Cell cycle arrest and apoptosis was tested by flow cytomety of propidium iodide stained cells after 24 hours of 1.0 μM Tam treatment. We found that the G1/G0 population increased 12.7 ±0.9% and 11.4±1.7% in the HMEC-LXSN and HMEC-cyto cells, respectively (Fig. 6A). Oneway ANOVA was performed and no significant difference was found between HMEC-LXSN and HMEC-cyto cells. Additionally, no increase in the sub-G1 population was observed in HMEC-LXSN or HMEC-cyto cells in response to Tam (data not shown). We used several additional approaches to determine whether Tam induced apoptosis in our HMEC models. First, we tested for caspase 3/7 activation using a fluorometric caspase activity assay. While we observed increased caspase activity in response to taxol, we did not observe increased caspase activity in HMECs treated with Tam for 24 hours (data not shown). Furthermore, the addition of the pan-caspase inhibitor Z-VAD-FMK did not inhibit Tam-induced cell death in HMECs when tested by MTT (Fig. 6B). To further rule out the possibility that Tam induced apoptosis in HMECs, we performed annexin V (AnV)/7-AAD staining on HMEC-control and HMEC-cyto cells. We found that Tam treatment for 24 hours resulted in a statistically significant decreases in viable cells (AnV-/7-AAD-) in both HMEC-LXSN and HMEC-cyto cells. A statistically significant difference in the Tam-treated AnV-/7-AAD- populations was observed between HMEC-LXSN and HMEC-cyto cells, with HMEC-cyto cells having more viable cells compared to HMEC-LXSN following Tam treatment. An increase in the AnV+/7-AAD+ (late apoptotic or necrotic) population was observed in both HMEC-LXSN and HMEC-cyto cells in response to Tam. Again, a statistically significant difference between HMEC-LXSN and HMEC-cyto Tam treated cells was observed (Fig. 6C). Fewer HMEC-cyto cells were AnV+/7-AAD+. A significant increase in the AnV+/7-AAD- population, indicative of early apoptosis, was not observed in either cell line at 24 hours or at an earlier 6 hour time point (data not shown). Taken together, the data presented in Fig. 6 suggests Tam induces apoptosis-independent cell death, which likely involves necrosis, and is inhibited by expression of PELP1-cyto.

Tam is known to induce autophagy [32,41,42]. Processing of LC3-I to LC3-II and subsequent accumulation of LC3-II is a marker of autophagy. To investigate the possibility that Tam treatment induced autophagy-associated necrosis in HMECs, we examined LC3-II levels in response to Tam in HMEC-LXSN, HMEC-PELP1-wt, and HMEC-PELP1-cyto cells by Western blot. Cell cultures were treated with increasing concentrations of Tam (1.0 and 2.0 μM) for 24 hours. Protein lysates were collected and separated by SDS-PAGE. Western blotting for LC3 revealed that Tam induced LC3-II accumulation in HMEC-LXSN and HMEC-PELP1-wt, but LC3-II accumulation was reduced in HMEC-PELP1-cyto cells compared to control cells (Fig. 6D).

To determine whether ERRγ expression has an effect on Tam-induced autophagy in HMECs, we examined LC3-II levels in response to Tam in MCF10A cells stably expressing vector control or ERRγ. MCF10A cells stably expressing ERRγ were more resistant to Tam-induced cell death (Fig. 4E). In cell cultures treated with increasing concentrations of Tam for 24 hours, we found that overexpression of ERRγ reduced Tam-induced LC3-II accumulation (Fig. 6E). We also examined Tam-induced LC3-II accumulation in HMEC-hTERTs expressing either shRNA control or ERRγ shRNA. Tam treatment of HMEC-LXSN cells expressing ERRγ shRNA resulted in a significant increase in LC3-II accumulation compared to HMEC-LXSN control cells. Similarly, ERRγ knockdown in HMEC-cyto cells also resulted in a significant increase in LC3-II accumulation in response to Tam (Fig. 6F). Together, the ERRγ overexpression and knockdown MTT data presented in Fig. 4 and the LC3 data presented in Fig. 6 indicate that PELP1-cyto and ERRγ expression suppresses Tam-induced LC3-II accumulation and autophagy-associated necrosis.

## Discussion

Herein, our study demonstrates a novel link between cytoplasmic PELP1 signaling, ERRγ, and cell survival in the presence of Tam. Our *in vivo* and *in vitro* findings suggest that PELP1 may be a useful biomarker of cell survival in response to Tam. PELP1 was localized to the cytoplasm in 36% of RPFNA samples from women at high-risk for developing breast cancer, and our *in vitro* findings show that cytoplasmic PELP1 localization promotes HMEC survival in response to Tam. Furthermore, we show that PELP1-cyto expression enhances ERRγ-dependent gene expression and that ERRγ expression promotes Tam resistance, in part, by blocking autophagy-associated necrosis. These data suggest an alternate mechanism of HMEC cell survival in the presence of Tam that could decrease the effectiveness of Tam chemoprevention.

### PELP1 and ERRγ as biomarkers of Tam resistance

The normal mammary gland is comprised of a heterogeneous population of cells, including luminal epithelial cells, basal myoepithelial cells, stromal cells, and immune cells. Relative to ER+ invasive breast cancer, which expresses high levels of ER, approximately 90% of normal mammary epithelial cells do not express ER and only 5–10% express moderate levels of ER [43]. Importantly, while the normal mammary gland expresses low ER levels compared to ER+ breast cancer, the normal mammary gland is clearly responsive to estrogens and Tam. Previously, we showed ER positivity in RPFNA samples from high-risk women predicts response to Tam chemoprevention [25]. Surprisingly, women with ER− RPFNA samples also responded to Tam chemoprevention. Thus, it is important to understand how both ER+ and ER− cells in high-risk breast tissue respond to Tam chemoprevention. Our data suggest that the presence of cytoplasmic PELP1 in high-risk epithelial cells could be an ER-independent biomarker that predicts survival of abnormal epithelial cells in the presence of Tam chemoprevention. There are limitations to the PELP1 IF results from RPFNA samples. The sample size is very small and currently none of the women whose RPFNA samples were tested have developed breast cancer in the five years in since the samples were tested. To test the hypothesis that cytoplasmic PELP1 promotes breast cancer initiation we would need to examine PELP1 localization in 300 samples to obtain 85% power to detect a difference in the incidence rate for a new breast cancer of 0.60 vs 0.44, assuming a one-sided alpha of 0.05. This calculation assumes that we will observe cytoplasmic PELP1 staining about 36% of the samples. Additionally, these women would need to be followed for up to 8 years to have 85% power to detect a difference in 3-year incidence rate for a new breast cancer of 26% vs 17%, assuming a one-sided alpha of 0.05. Further evidence supporting a role for PELP1 as a biomarker of breast cancer initiation would provide a strong rationale for these larger and longer-term studies.

### PELP1 and ERRγ both function to promote Tam-resistance

PELP1 is a large multi-domain protein that modulates many cell signaling pathways and biological processes. PELP1 was first identified as an ER co-activator protein and has since been shown to function as a transcriptional co-activator and co-repressor for several nuclear receptors and other transcription factors. PELP1 is frequently overexpressed in breast cancer [44], and altered localization to the cytoplasm has been described in approximately 50% of PELP1-positive breast tumors [17,18]. Vadlamudi and colleagues were the first to demonstrate altered PELP1 localization in human tumors and show that cytoplasmic PELP1 signaling promotes Tam resistance in ER+ breast cancer cell line models [17,18,45]. MCF-7 cells stably expressing PELP1-cyto had increased activation of Akt and Erk1/2 signaling, which promoted phosphorylation of ER at serines 167 and 118, respectively. While PELP1-induced signaling to Erk1/2 was dependent on the presence of ER, Akt activation was independent of ER expression [17]. PELP1-cyto signaling to Akt and Erk1/2 was confirmed in the MMTV-PELP1-cyto transgenic mouse model [18]. These mice formed widespread epithelial hyperplasia, which was resistant to Tam treatment. Interestingly, in primary HMECs Tam inhibits Akt serine 473 phosphorylation, which promotes Tam-induced cell death [27]. Overexpression of constitutively active Akt was shown to block Tam-induced cell death in this model. Thus, we hypothesized that PELP1-cyto-induced Akt activation, independent of ER, would inhibit Tam-induced cell death in our immortalized HMEC models. We found that PELP1-cyto does in fact protect HMECs from Tam-induced cell death, but independent of Akt activation. As Erk1/2 activation also plays a role in Tam-resistance in ER+ breast cancer, we also tested our models for Erk1/2 activation. Similar to our Akt studies, we did not observe a significant increase in Erk1/2 activation in our cell line models. The modest effect of Akt and MEK inhibitors on Tam-induced cell death suggests that other signaling mechanisms are likely to be more important.

Similar to PELP1, ERRγ promotes breast cancer progression both *in vivo* and *in vitro*. *In vivo*, ERRγ expression is correlated with ER expression [12]. While this was initially interpreted as ERRγ being a favorable prognostic indicator, subsequent studies have found ERRγ expression to be associated with positive lymph node status [46] and an increased risk of recurrence in ER+ breast cancer [14,38]. *In vitro* studies support the *in vivo* observation, where it has been found that ERRγ promotes Tam resistance in ER+ breast cancer cell line models [13,14]. Tam-resistant SUM44 invasive lobular carcinoma cells (LCCTam) were found to upregulate ERRγ expression. ERRγ knockdown in LCCTam cells resulted in enhanced sensitivity to Tam, while ERRγ overexpression promoted Tam resistance in the parental SUM44 cell line [13]. Riggins and colleagues went on to show that Erk signaling stabilizes ERRγ protein levels via phosphorylation of specific serine residues, which contributes to ERRγ-induced Tam resistance in MCF-7 and SUM44 cell lines [14]. Our studies in HMECs compliment these previous reports. Overexpression of ERRγ enhanced cell survival, while ERRγ knockdown sensitized PELP1-cyto-expressing HMECs to Tam-induced cell death (Fig. 4). Because our cell line models do not express ER, our studies suggest that mechanistically ERRγ contributes to Tam-resistance independent of ER signaling. Additionally, our data suggest that inhibition of ERRγ will sensitize ER-low pre-malignant or atypical mammary epithelial cells expressing cytoplasmic PELP1 to Tam chemoprevention.

### PELP1 and ERRγ signaling mediate resistance to Tam-induced autophagy associated necrosis

Tam’s actions have primarily been studied in ER+ breast cancer cell line models *in vitro* and *in vivo*. In ER+ breast cancer cell line models, Tam promotes cell cycle arrest, apoptosis, and autophagy [34,36]. Early studies found that treatment of cells with Tam, 4-OH-Tam, and other anti-estrogens resulted in ER-dependent G1 cell cycle arrest (reviewed in [34]). Interestingly, Tam’s ER-independent effects have been observed in both ER+ and ER− cell lines. In most of the studies describing ER-independent effects, Tam induced apoptosis and/or autophagy at 5–10 μM concentrations of Tam. In our immortalized HMEC lines, we observed Tam-induced autophagy starting at 0.25 μM and complete toxicity at 2 μM, well below therapeutic tissue concentrations achieved *in vivo*. Similar to other reports in ER− cells lines [32], our results suggest that Tam-induced cell death is dependent on autophagy-associated necrosis.

While PELP1 was previously shown to localize to autophagosomes [47,48], the work presented here is the first to show a definitive role for PELP1 and ERRγ in autophagy. Prior reports suggest that the PELP1-interacting protein, HRS, promotes PELP1 autophagosome localization in response to resveratrol. The authors suggest that resveratrol-induced autophagy selectively results in PELP1 degradation to promote the inhibitory effects of resveratrol, but knockdown or overexpression of PELP1 was not performed to determine whether PELP1 expression affects cell proliferation or survival in the presence of resveratrol. Interestingly, we did not observe changes in PELP1 protein levels after Tam treatment or co-localization of PELP1 and LC3 following Tam treatment; this suggests that Tam-induced autophagy does not target PELP1 for degradation (data not shown). The results presented here indicate that PELP1-cyto, but not wild-type PELP1, promotes cell survival in the presence of an autophagy-inducing stimulus. Further studies are needed to determine whether enhanced cell survival in the presence of PELP1-cyto is specific to Tam or can be expanded to other autophagy inducing compounds.

The predominate transcriptional targets of ERRs are involved in metabolism and mitochondrial biogenesis (reviewed in [11]). It is therefore surprising—given the intimate connection between nutrient conditions, cell metabolism, and autophagy [49]—that a connection between ERRs and autophagy has not been described. Our data clearly show that overexpression of ERRγ inhibits LC3-II accumulation in response to Tam, while ERRγ knockdown enhances Tam-induced LC3-II accumulation (Fig. 6). While we have not identified the ERRγ-dependent molecular mechanisms or gene targets associated with the autophagy response, it is likely that future global gene expression studies will help unravel these questions. Interestingly, indirect evidence for a link between ERRγ and autophagy does exist. Hypoxia induces autophagy in the tumor microenvironment to promote cell survival and tumorigenesis [50]. Interestingly, hypoxia also induces ERRγ expression and ERRγ-dependent gene expression [51]. This study did not examine the effect of ERRγ overexpression or knockdown in response to hypoxia, so it is unknown how ERRγ expression affects hypoxia-induced cell death or if this is associated with enhanced autophagy.

### Future Directions and Conclusion

Our study is the first to report a link between PELP1-cyto signaling and ERRγ. Indeed, PELP1 has been shown to interact with, and act as a transcription co-regulator for many nuclear receptors including ERRα (reviewed in [52] and [53]). Although possible, our data does not suggest a direct interaction between PELP1 and ERRγ. Instead, we propose that PELP1-induced signaling in the cytoplasm promotes activation of ERRγ and specific ERRγ-dependent gene expression to promote cell survival in response to Tam. We show that PELP1-cyto induces expression of MAOB, a known ERRγ-dependent gene, and that ERRγ regulates a known PELP1-regulated gene, MMP-3. As inhibitors of MAOB (rasagiline mesylate) and MMP-3 (UK-356618) are available, we tested these compounds in MTT assays to determine whether they sensitize PELP1-cyto cells to Tam. Unfortunately, neither compound sensitized PELP1-cyto expressing cells to Tam (data not shown). Therefore, it is likely that other PELP-cyto-induced, ERRγ-dependent genes exist and play a role in response to Tam chemoprevention. Global gene expression analysis of PELP1-cyto/ERRγ knockdown cells will likely reveal novel gene targets important for response to autophagy-inducing compounds.

In conclusion, our data support a model in which altered PELP1 localization to the cytoplasm during early mammary carcinogenesis has the potential to alter response to Tam chemoprevention. The molecular mechanism involves cytoplasmic PELP1 signaling that promotes ERRγ-dependent gene expression, which functions to promote cell survival in the presence of Tam chemoprevention. We hypothesize that targeting PELP1, PELP1 downstream signaling, or ERRγ would promote Tam-induced cell death in atypical mammary epithelial cells. Thus, PELP1 localization, and/or ERRγ activation could be developed as a biomarker for response to Tam chemoprevention and could be targeted to enhance the efficacy of Tam chemoprevention.
